# Job Strain and Cardiovascular Disease Risk Factors: Meta-Analysis of Individual-Participant Data from 47,000 Men and Women

**DOI:** 10.1371/journal.pone.0067323

**Published:** 2013-06-20

**Authors:** Solja T. Nyberg, Eleonor I. Fransson, Katriina Heikkilä, Lars Alfredsson, Annalisa Casini, Els Clays, Dirk De Bacquer, Nico Dragano, Raimund Erbel, Jane E. Ferrie, Mark Hamer, Karl-Heinz Jöckel, France Kittel, Anders Knutsson, Karl-Heinz Ladwig, Thorsten Lunau, Michael G. Marmot, Maria Nordin, Reiner Rugulies, Johannes Siegrist, Andrew Steptoe, Peter J. M. Westerholm, Hugo Westerlund, Töres Theorell, Eric J. Brunner, Archana Singh-Manoux, G. David Batty, Mika Kivimäki, for the IPD-Work Consortium

**Affiliations:** 1 Finnish Institute of Occupational Health, Helsinki and Tampere, Finland; 2 School of Health Sciences, Jönköping University, Jönköping, Sweden; 3 Institute of Environmental Medicine, Karolinska Institutet, Stockholm, Sweden; 4 Stress Research Institute, Stockholm University, Stockholm, Sweden; 5 Centre for Occupational and Environmental Medicine, Stockholm County Council, Stockholm, Sweden; 6 School of Public Health, Université libre de Bruxelles, Brussels, Belgium; 7 Department of Public Health, Ghent University, Ghent, Belgium; 8 Institute for Medical Sociology, Medical Faculty, University of Düsseldorf, Düsseldorf, Germany; 9 Department of Cardiology, West-German Heart Center Essen, University Duisburg-Essen, Essen, Germany; 10 School of Community and Social Medicine, University of Bristol, Bristol, United Kingdom; 11 Department of Epidemiology and Public Health, University College London, London, United Kingdom; 12 Institute for Medical Informatics, Biometry, and Epidemiology, Faculty of Medicine, University Duisburg-Essen, Essen, Germany; 13 Department of Health Sciences, Mid Sweden University, Sundsvall, Sweden; 14 German Research Center for Environmental Health, Neuherberg, Germany; 15 Department of Psychology, Umeå University, Umeå, Sweden; 16 National Research Centre for the Working Environment, Copenhagen, Denmark; 17 Department of Public Health and Department of Psychology, University of Copenhagen, Copenhagen, Denmark; 18 Occupational and Environmental Medicine, Uppsala University, Uppsala, Sweden; 19 Inserm U1018, Centre for Research in Epidemiology and Population Health, Villejuif, France; 20 Centre for Cognitive Ageing and Cognitive Epidemiology, University of Edinburgh, Edinburgh, United Kingdom; 21 Institute of Behavioral Sciences, University of Helsinki, Helsinki, Finland; Istituto Clinico S. Ambrogio, Italy

## Abstract

**Background:**

Job strain is associated with an increased coronary heart disease risk, but few large-scale studies have examined the relationship of this psychosocial characteristic with the biological risk factors that potentially mediate the job strain – heart disease association.

**Methodology and Principal Findings:**

We pooled cross-sectional, individual-level data from eight studies comprising 47,045 participants to investigate the association between job strain and the following cardiovascular disease risk factors: diabetes, blood pressure, pulse pressure, lipid fractions, smoking, alcohol consumption, physical inactivity, obesity, and overall cardiovascular disease risk as indexed by the Framingham Risk Score. In age-, sex-, and socioeconomic status-adjusted analyses, compared to those without job strain, people with job strain were more likely to have diabetes (odds ratio 1.29; 95% CI: 1.11–1.51), to smoke (1.14; 1.08–1.20), to be physically inactive (1.34; 1.26–1.41), and to be obese (1.12; 1.04–1.20). The association between job strain and elevated Framingham risk score (1.13; 1.03–1.25) was attributable to the higher prevalence of diabetes, smoking and physical inactivity among those reporting job strain.

**Conclusions:**

In this meta-analysis of work-related stress and cardiovascular disease risk factors, job strain was linked to adverse lifestyle and diabetes. No association was observed between job strain, clinic blood pressure or blood lipids.

## Introduction

Psychological stress at work, or job strain, has been shown to be moderately associated with an increased risk of coronary heart disease [1]–[4]. However, despite a series of studies, the association of this psychological characteristic with many cardiovascular risk factors remains unclear. While there is evidence that stress is linked to unfavourable levels of lifestyle factors, such as physical activity, smoking habits, alcohol consumption and weight control [5]–[8], its influence, if any, on biological risk factors, especially clinic blood pressure, blood lipids and blood glucose, remains controversial [9]–[18]. Many studies of stress biology are characterised by small sample sizes, single risk factor outcomes, and the use of heterogeneous measures of stress. If we are to understand risk management in people with job strain, larger studies which capture a wide range of risk factors are needed._ENREF_17 Accordingly, we conducted the largest study on this issue to date by pooling individual-level data from eight European studies comprising a total of 47,045 men and women.

## Materials and Methods

### Study population

We used data from eight independent studies, in which clinical examinations had been conducted between 1984 and 2003, in Belgium (Belstress [19]), Germany (HNR [20], KORA S1-S3 [21]), Sweden (WOLF-N [13], WOLF-S [22]) and the UK (Whitehall II [23]). All studies are part of the "Individual-Participant-Data Meta-analysis of Working Populations" (IPD-Work) Consortium established in 2008 [4]. Ethical approval for each constituent study in the IPD-Work consortium was obtained from the relevant local or national ethics committees and all participants gave informed consent to take part. Details of the design, recruitment, and ethical approval for the participating studies are described elsewhere and presented in Text S1.

Our analyses were based on 47,045 participants who were in employment at the time of the assessment and underwent a clinical examination. We excluded 4394 (8.5%) participants with missing information on sex, age, or job strain, or with a history of myocardial infarction (data on prevalent myocardial infarction was not available from KORA).

### Assessment of job strain

Job strain was measured in all studies using questions from the Job Content Questionnaire and Demand-Control Questionnaire [24]. Briefly, enquiries were made about the psychosocial aspects of study members’ job. For each participant, mean response scores were calculated for job demands items and job control items. High job demands were defined as a score in this domain that was higher than the study-specific median score; low job control was defined as a score in this domain that was lower than the study-specific median score. Job strain was then denoted by high demands and low control and, for the purposes of analyses, compared to all other combinations (no strain). We have previously published a detailed description of this job strain measure, including its validation and harmonization, as part of this collaboration [25].

### Assessment of demographic characteristics

Socioeconomic status (SES) was based on occupational position obtained from employers' or other registers, or participant-completed questionnaires. SES was categorized into low, intermediate or high. Participants who were self-employed or who had missing data on job title were included in the analyses in the "other" SES category. We also identified respondents who worked in shifts.

### Assessment of cardiovascular disease risk factors

Participants underwent a clinical examination where their height, weight, and blood pressure were measured; a blood sample was also taken. Body mass index (BMI) was calculated as weight in kilograms divided by height in meters squared and, based on World Health Organization (WHO) guidance, obesity was defined as a BMI of ≥ 30 kg/m^2^ [26]. Hypertension was denoted by as systolic (diastolic) blood pressure of at least 140 (90) mmHg, or use of antihypertensive medication. Total and HDL-cholesterol levels were measured in all studies, but triglyceride values were only available in four (HNR, WOLF N, WOLF S and Whitehall II). Blood cholesterol ratio was defined as the total divided by HDL cholesterol. Diabetes and the use of antihypertensive or lipid-lowering medication were based on self-report. In the Whitehall II study, diabetes was additionally measured by 2-h oral glucose tolerance test [27]. In addition to these standard risk factors, we assessed pulse pressure, computed as systolic minus the diastolic blood pressure, because high pulse pressure is an independent correlate of atherosclerosis [28].

We extracted data on smoking, alcohol use, and physical inactivity from standard questionnaires completed by participants in all studies. While there were inevitably some differences in the questions used to ascertain levels of smoking, alcohol intake and physical activity across studies, we were able to harmonise these data [5]–[7]. In general, the enquiries used are standard and have shown sufficiently high agreement with objective measures of these behaviours to justify their use in large, population-based surveys [29]–[32]. Smoking status was dichotomized (current smoker or non-smoker) [6]. Alcohol use was requested by questions on the total number of alcoholic drinks, by type of drink, which the participants consumed in a week. One drink was defined as approximately equivalent to one unit or one glass of alcoholic drink or 10 g of ethanol. Alcohol use was categorized as none, moderate use (1–15 and 1–22 units of alcohol per week in women and men, respectively) or greater [7]. The questions used to assess leisure-time physical activity differed between studies. Some studies had only questions on sports activities and exercise, while for other studies information was also available for other types of leisure-time physical activities, such as walking and cycling. Participants were denoted as being physically inactive if they reported none or very little moderate or vigorous leisure-time physical activity or exercise [5]. As expected, smoking and physical inactivity were associated with incident coronary heart disease in IPD-Work [33].

To assess overall cardiovascular disease risk, we constructed the Framingham cardiovascular disease risk score on the basis of age, total cholesterol, HDL cholesterol, systolic blood pressure, hypertensive medication use, smoking and diabetes status. Following clinical guidelines, “high” overall risk was defined as a Framingham score of 20% or higher [34].

### Data analysis

Individual-level data from the studies were pooled into one dataset. The associations between job strain and CVD risk factors were analyzed using mixed effects linear and logistic regression models with the study as the random effect. In these analyses, triglyceride values were logarithmically transformed due to their skewed distribution. Measures of association were adjusted for sex and age, and additionally for SES. In the main analysis, we excluded participants who reported use of antihypertensive medication when the outcome was diastolic or systolic blood pressure or pulse pressure, and participants who reported the use of lipid-lowering medication when the outcome was any measure of cholesterol or triglycerides although a sensitivity analysis was conducted including these participants. In further analyses of statistically significant job strain-risk factor associations, multiple multivariable adjustments were undertaken to examine the robustness of each association. Because shift or night time work has been found to be a strong predictor of the metabolic syndrome [35], the job strain-diabetes association was repeated excluding participants who had shift or night time work. SAS statistical software, version 9.2, was used for all statistical analyses.

## Results

The basic characteristics of the participants according to each study are presented in **Table 1**. Mean age was 45.1 years and 29.2% of the study members were women. **Table 2** shows age- and sex-adjusted associations between job strain and various risk factors. Compared to participants without job strain, those reporting job strain were 35% more likely to have diabetes (odds ratio 1.35, 95% confidence interval 1.15, 1.57). These associations were little changed after additional adjustment for SES. Job strain was associated with several lifestyle variables, such as physical inactivity (1.43, 95% CI 1.36, 1.51), current smoking (1.23, 95% CI 1.16, 1.30), alcohol abstinence (1.21, 95% CI 1.13, 1.30) and obesity (1.19, 95% CI 1.11, 1.28).

**Table 1:** Participant Characteristics According to Study, the IPD-Work Consortium, 1984–2003.

Study	Baseline	N	Age (SD), y	Women (%)	Job strain (%)
Belstress [19]	1994–1998	20,692	45.4 (5.9)	4909 (23.7)	3900 (18.9)
Heinz-Nixdorf Recall [20]	2000–2003	1776	53.3 (4.8)	736 (41.4)	217 (12.2)
KORA Survey 1 [21]	1984–1985	2460	42.3 (10.2)	864 (35.1)	483 (19.6)
KORA Survey 2 [21]	1989–1990	2370	42.3 (10.6)	896 (37.8)	417 (17.6)
KORA Survey 3 [21]	1994–1995	2345	42.6 (10.4)	953 (40.6)	372 (15.9)
WOLF Norrland [13]	1996–1998	4678	44.0 (10.3)	780 (16.7)	599 (12.8)
WOLF Stockholm [22]	1992–1995	5654	41.5 (11.0)	2447 (43.3)	917 (16.2)
Whitehall II [23]	1991–1993	7070	48.8 (5.7)	2168 (30.7)	959 (13.6)
Pooled data	1984–2003	47,045	45.1 (8.4)	13,753 (29.2)	7864 (16.7)

**Table 2:** Association Between Job Strain and Biological and Lifestyle Risk Factors, the IPD-Work Consortium, 1984–2003.

		Mean (SE) §			
	Total N	No strain	Job strain	Mean difference (95% CI)§	Mean difference (95% CI)#
**Biological risk factors**					
Systolic blood pressure, mmHg*	44,106	126.8 (1.6)	126.8 (1.6)	0.01 (–0.35, 0.38)	–0.01 (–0.38, 0.36)
Diastolic blood pressure, mmHg*	44,104	79.5 (1.1)	79.5 (1.1)	–0.04 (–0.28, 0.21)	0.01 (–0.24, 0.26)
Pulse pressure, mmHg *	44,104	47.3 (1.2)	47.3 (1.2)	0.05 (–0.21, 0.31)	–0.02 (–0.28, 0.24)
Total cholesterol, mmol/l †	45,776	5.87 (0.1)	5.89 (0.1)	0.01 (–0.01, 0.04)	0.01 (–0.02, 0.04)
HDL, mmol/l †	45,728	1.42 (0.01)	1.41 (0.01)	–0.01 (–0.02, –0.00)	–0.001 (–0.01, 0.01)
Cholesterol ratio †	45,723	4.5 (0.1)	4.6 (0.1)	0.04 (0.00, 0.09)	0.01 (–0.03, 0.06)
Triglycerides, mmol/l †	18,858	1.4 (0.1)	1.4 (0.1)	0.01 (–0.01, 0.04)	–0.001 (–0.02, 0.02)
		**Prevalence (%)** §		**Odds ratio (95% CI)** §	**Odds ratio (95% CI)#**
Hypertension	47,045	30.4	30.1	0.99 (0.94, 1.05)	0.99 (0.93, 1.04)
Diabetes	46,510	2.2	2.8	1.35 (1.15, 1.57)	1.29 (1.11, 1.51)
**Lifestyle risk factors**					
Smoking	46,553	26.6	30.7	1.23 (1.16, 1.30)	1.14 (1.08, 1.20)
Non-drinking	46,482	16.5	19.3	1.21 (1.13, 1.30) ‡	1.11 (1.04, 1.19)
High alcohol use	46,482	21.6	21.6	1.06 (0.99, 1.13) ‡	1.06 (0.99, 1.14)
Physical inactivity	46,395	31.7	38.7	1.43 (1.36, 1.51)	1.34 (1.26, 1.41)
Obesity	46,891	13.7	15.7	1.19 (1.11, 1.28)	1.12 (1.04, 1.20)
**Overall cardiovascular risk**					
Framingham risk ≥20	45,428	9.6	9.9	1.19 (1.08, 1.31)	1.13 (1.03, 1.25)

* Participants not using antihypertensive medication.

† Participants not using lipid-lowering medication.

‡ Compared to moderate drinkers only.

§ Age- and sex-adjusted.

#Age-, sex-, and SES-adjusted.

**Table 2** also shows that in the age-, sex-, and SES-adjusted analyses, there were no differences between people with and without job strain for systolic or diastolic blood pressure, pulse pressure, cholesterol or triglyceride values. These findings were unchanged in sensitivity analyses: No associations of job strain with blood pressure and blood cholesterol were observed after including participants treated with antihypertensive and lipid-lowering drugs in the analysis: age-, sex- and SES-adjusted mean difference in systolic blood pressure and total cholesterol 0.08 (95% confidence interval –0.29, 0.45) mmHg and 0.01 (95% confidence interval –0.01, 0.04) mmol/L between those with and without job strain, respectively. This was also the case after adding a constant of 10 mmHg to systolic blood pressure values among participants on antihypertensive treatment (adjusted difference 0.12, 95% confidence interval –0.26, 0.50 mmHg) and a constant of 2 mmol/L to total cholesterol values among participants on lipid-lowering treatment (adjusted difference 0.01, 95% confidence interval –0.01, 0.04 mmol/L) (N  =  46,991 and 46,659 in these analyses) [36].

Job strain was associated with a slightly higher overall cardiovascular disease risk (1.19, 95% CI 1.08, 1.31), as indicated by a Framingham risk ≥20%; this association was due to the higher prevalence of physical inactivity, smoking and diabetes among participants with job strain (odds ratio after adjusting for these factors: 1.03, 95% CI 0.92, 1.16).

**Figure 1:**
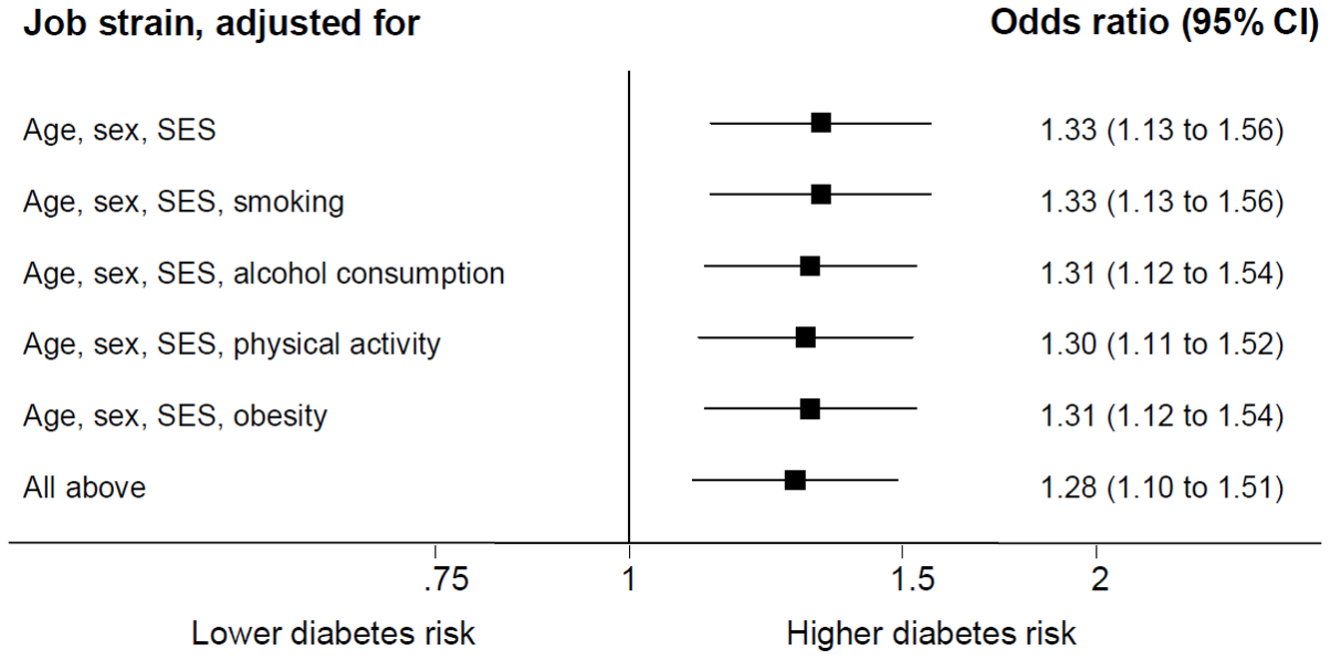
Adjusted Odds Ratios (95% CI) for the Association Between Job Strain and Diabetes (N  =  44,818 in All Models), the IPD-Work Consortium, 1984–2003.

In **figure 1** we present results from the multivariable adjusted analyses for the job strain-diabetes association. The age-, sex- and SES-adjusted association was little attenuated after additional adjustment for smoking, alcohol consumption, physical inactivity and obesity, suggesting that the association is not explained by lifestyle factors.Sex-specific analyses showed little difference in the associations of job strain with diabetes (age- and SES-adjusted odds ratio 1.21, 95% CI 1.00, 1.46 in men and 1.48, 95% CI 1.12, 1.97 in women). No significant interaction effect between sex and job strain was found for diabetes (P = 0.18) either. The exclusion of the shift and night workers only slightly attenuated the age-, sex and SES-adjusted odds ratio (1.20, 95% CI 0.99, 1.45).

## Discussion

Meta-analysis of individual participant data from over 47,000 participants showed that persons with job strain had higher prevalence of diabetes. This association was robust to adjustment for smoking, alcohol consumption, physical inactivity and obesity, suggesting that it is not explained by poorer lifestyle among persons reporting job strain. Contrary to popular opinion, we found no clinically relevant differences in lipid levels, clinic blood or pulse pressure or prevalence of hypertension between participants with or without job strain.

We used a pre-defined measure of job strain which was harmonised before the inclusion and analysis of risk factors, excluding bias arising from *post hoc* exposure definition [25]. Furthermore, the job strain measure has been shown to be associated with subsequent coronary heart disease in this dataset [4], suggesting that imprecise measurement, present when capturing any self-reported variable, is an unlikely explanation for the absence of associations with some of the biological factors. The associations with lifestyle factors in this analysis of 8 studies with biological data corresponded to our findings reported for the entire IPD-Consortium of >140,000 men and women [5]–[8]. A limitation of our study is that it is not based on a systematic review of all available data in the field. Also, we cannot draw causal inference due to the non-randomised nature of the utilised data. These points notwithstanding, a causal association is unlikely if no cross-sectional association is observed.

The key mediators of the association between job strain and cardiovascular diseases have long been debated. Our findings are in agreement with the view that job strain affects disease risk via poor health behaviors, and by increasing risk of diabetes. A previous report using longitudinal data from the IPD-Work consortium has shown that job strain predicts physical inactivity more strongly than physical inactivity predicts job strain [5]. This is consistent with the expected causal direction of the association. The evidence on the temporal nature of the association between job strain, obesity, smoking and drinking patterns is less clear. It is possible that the associations are bidirectional and partially explained by common causes [6]–[8].

An alternative hypothesis is that job strain affects the development of cardiovascular diseases by directly altering standard biological risk factors. Our analyses provide limited support for this view as we found no evidence of a consistent association between job strain and most of the cardiovascular disease risk factors. Our findings of the absence of a relation between resting blood pressure and hypertension are concordant with several previous studies in this field [13], [16], [37], although this is not a universal finding and does not apply to findings on ambulatory blood pressure [9], [10], [38], [39]. Similarly, the present results are in agreement with earlier studies which have concluded that job strain is not associated with cholesterol [13], [37], [38], [40], although, again, the literature is discordant [11], [41].

Our results show job strain to be related to increased risk of diabetes. This association was present in age- and sex-adjusted models, and after adjustment for SES and measures of health behavior. Furthermore, the association between job strain and diabetes was somewhat stronger in women than men, in accordance with other evidence [18], [42], [43]. Our findings support the possibility that job strain contributes to disturbances in glucose metabolism leading to a raised risk of diabetes. However, given the cross-sectional nature of these data, we cannot exclude the possibility that a chronic condition, such as diabetes, affected perceptions of job strain.

In principle, stress could simultaneously affect multiple risk factors, rather than a particular risk factor, and thus increase the risk of cardiovascular diseases. To test this possibility, we assessed the overall risk using a validated multifactorial risk algorithm, the Framingham score, comprising age, total cholesterol, HDL-cholesterol, systolic blood pressure, hypertensive medication use, smoking, and diabetes status [34], [44]. We found job strain to be associated with elevated Framingham risk, although this association was attributable to the combination of poor lifestyle and increased diabetes prevalence among those with job strain.

These results suggest that job strain links to cardiovascular disease risk mostly via lifestyle factors and hyperglycemia. Our findings provide strong evidence against the common belief that job strain increases resting blood pressure. Similarly, we found no evidence to suggest that job strain is associated with pulse pressure. However, there is a range of other potential biological stress mediators to be assessed in future studies: chronic inflammation (e.g., interleukin 6) [45], blood coagulation factors, and increased risk of stress response that act as a trigger of cardiac events among individuals with undiagnosed advanced atherosclerosis. It has also been suggested that non-dipping blood pressure is more prevalent among individuals with job strain [46], [47].

Our findings are based on a large number of participants, providing sufficient power to detect relatively small effects and also to confirm the absence of an association. The study covers a wide range of risk factors and a measure of overall cardiovascular risk; and it is the first to use an individual participant meta-analysis methodology to examine the association between job strain and risk factors. These data suggest that risk management among people with job strain should focus on glucose levels and lifestyle factors. The main emphasis of future mechanistic investigations of job strain and cardiovascular disease risk should be placed on examining diabetes and lifestyle factors rather than standard cardiovascular risk factors.

## Supporting Information

Text S1 **Studies and participants.**
(DOC)

Checklist S1 **PRISMA Checklist.**
(DOC)
