# Supplementary material for: Job Strain and Cardiovascular Disease Risk Factors: Meta-Analysis of Individual-Participant Data from 47,000 Men and Women
Source: PLoS One. 2013 Jun 20;8(6):e67323. doi: 10.1371/journal.pone.0067323 (PMC3688665; doi:10.1371/journal.pone.0067323)
Supplement: Text S1 — Studies and participants. (DOC) [file pone.0067323.s001.doc]

**Text S1. Studies and participants**

Details of the design and recruitment of the participants in the studies included in our meta- analyses are presented below. Participants were eligible for our meta-analyses if they were in employment and had available data on job strain.

**Belstress**

Belstress is a prospective cohort study set up to investigate the associations between work-related stress and health outcomes. Between 1994 and 1998, 21 419 people aged 35-59 were recruited into the study from the payroll records of 25 large companies or public administrations.^1 2^ Of these, 21 024 men and women had data on job strain and were eligible for our meta-analyses. The ethics committees of the University Hospital of Ghent and the Faculty of Medicine of the Université Libre de Bruxelles approved the Belstress study.

**Heinz Nixdorf Recall study (HNR)**

The Heinz Nixdorf Recall Study is a prospective population-based cohort study of individuals randomly selected from the mandatory lists of residence in the metropolitan Ruhr area in Germany. Details of the study methods have been described previously.^3 4^ Briefly, 4 814 participants aged 45-75 years were enrolled at study baseline in 2000-2003. Job stress measures and comprehensive medical data were collected during the baseline examination. For the present analyses baseline job strain measures were available for 1 841 employed men and women. The HNR was approved by the institutional local ethical committees and a quality management system according to European industrial norms (DIN EN ISO 9001:2000) was applied

**Cooperative Health Research in the Region Augsburg (KORA S1-S3)**

KORA is a regional research platform for population-based surveys and subsequent follow-up studies in the fields of epidemiology, health economics, and health care research. KORA was established in 1996 to continue and expand the MONICA project in Augsburg, including the Acute Myocardial Infarction (AMI) Registry. KORA stands for "Kooperative Gesundheitsforschung in der Region Augsburg" (Cooperative Health Research in the Region of Augsburg) and incorporates two important characteristics: the population based research design on a regional basis and the cooperative research structure. The study region of Augsburg has a population of about 600,000 of which 430,000 inhabitants are between 25 and 74 years of age. Cross-sectional health surveys were performed in the population aged 25 to 74 with German nationality. Samples were drawn in a two-stage procedure where first Augsburg city and sixteen communities from the adjacent counties were selected by cluster sampling and then stratified random sampling was performed within each community. In this way, three cross-sectional health surveys S1 to S3 have been performed at five year intervals (1984/1985, 1989/1990, and 1994/1995), each survey comprising an independent random sample. In total, 13 818 men and women participated in S1 to S3.^5^ The Ethics Committee of the Bavarian Medical Association and the Bavarian commissioner for data protection and privacy approved the study.

**Whitehall II**

The Whitehall II study is a prospective cohort study set up to investigate socioeconomic determinants of health. At study baseline in 1985-1988, 10 308 civil service employees (6 895 men and 3 413 women) aged 35-55 and working in 20 civil service departments in London were invited to participate in the study.^6^ Data on job strain, measured at study baseline were available for 10 285 men of the men and women who were eligible for our meta-analyses. The Whitehall II study protocol was approved by the University College London Medical School committee on the ethics of human research. Written informed consent was obtained at each data collection wave.

**WOLF (Work, Lipids, and Fibrinogen) Stockholm and WOLF Norrland studies**

The WOLF (Work, Lipids, and Fibrinogen) Stockholm study is a prospective cohort study of 5 698 people (3 239 men and 2 459 women) aged 19–70 and working in companies in Stockholm county.^7^ WOLF Norrland is a prospective cohort of 4 718 participants aged 19-65 working in companies in Jämtland and Västernorrland counties.^8^ At study baseline the participants underwent a clinical examination and completed a set of health questionnaires. For WOLF Stockholm, the baseline assessment was undertaken at 20 occupational health units between November 1992 and June 1995 and for WOLF Norrland at 13 occupational health service units in 1996-98. The Regional Research Ethics Board in Stockholm, and the ethics committee at Karolinska Institutet, Stockholm, Sweden approved the study.

**References**

1. Pelfrene E, Vlerick P, Mak RP, De Smets P, Kornitzer M, De Backe G. Scale reliability and validity of the Karasek `Job Demand-Control-Support’ model in the Belstress study. *Work & Stress,* 2001;15(4):297-313.

2. De Bacquer D, Pelfrene E, Clays E, Mak R, Moreau M, de Smet P, et al. Perceived job stress and incidence of coronary events: 3-year follow-up of the Belgian Job Stress Project cohort. *Am J Epidemiol* 2005;161(5):434-41.

3. Schmermund A, Mohlenkamp S, Stang A, Gronemeyer D, Seibel R, Hirche H, et al. Assessment of clinically silent atherosclerotic disease and established and novel risk factors for predicting myocardial infarction and cardiac death in healthy middle-aged subjects: rationale and design of the Heinz Nixdorf RECALL Study. Risk Factors, Evaluation of Coronary Calcium and Lifestyle. *Am Heart J* 2002;144(2):212-8.

4. Stang A, Moebus S, Dragano N, Beck EM, Mohlenkamp S, Schmermund A, et al. Baseline recruitment and analyses of nonresponse of the Heinz Nixdorf Recall Study: identifiability of phone numbers as the major determinant of response. *Eur J Epidemiol* 2005;20(6):489-96.

5. Holle R, Happich M, Lowel H, Wichmann HE. KORA--a research platform for population based health research. *Gesundheitswesen* 2005;67 Suppl 1:S19-25.

6. Marmot MG, Smith GD, Stansfeld S, Patel C, North F, Head J, et al. Health inequalities among British civil servants: the Whitehall II study. *Lancet* 1991;337(8754):1387-93.

7. Peter R, Alfredsson L, Hammar N, Siegrist J, Theorell T, P. W. High effort, low reward, and cardiovascular risk factors in employed Swedish men and women: baseline results from the WOLF Study. *J Epidemiol Community Health* 1998;52:540-47

8. Alfredsson L, Hammar N, Fransson E, de Faire U, Hallqvist J, Knutsson A, et al. Job strain and major risk factors for coronary heart disease among employed males and females in a Swedish study on work, lipids and fibrinogen. *Scand J Work Environ Health* 2002;28(4):238-48.
